# Peer Review of “Medical Expectations of Physicians on AI Solutions in Daily Practice: Cross-Sectional Survey Study”

**DOI:** 10.2196/56446

**Published:** 2024-03-25

**Authors:** Lucía Carrasco-Ribelles

**Affiliations:** 1 Fundació Institut Universitari per a la recerca a l'Atenció Primària de Salut Jordi Gol i Gurina, Barcelona, Spain

**Keywords:** artificial intelligence, adoption, acceptance, opinion, perceptions, survey, expectations, physician, medical survey, qualitative study, AI

*This is the peer-review report for “Medical Expectations of Physicians on AI Solutions in Daily Practice: Cross-Sectional Survey Study.”*

## Round 1 Review

### General Comments

This paper [1] reports the results of a survey on medical expectations on artificial intelligence (AI) solutions in daily practice. The authors argue that it is important to know the opinion that physicians would have as users of these solutions, and the reviewer could not agree more. Therefore, the results of this work may be of interest to the community.

### Specific Comments

#### Major Comments

1. The authors say that these results represent the opinion of Brazilian physicians. Perhaps that is a bit presumptuous, at least without somehow justifying the size of the hospital relative to the Brazilian population. What percentage of the Brazilian population attends this hospital? What percentage of Brazilian physicians works there?

2. I have not been able to find the supplementary material anywhere. Therefore, I could not review the complete questionnaire.

3. The division into <20 years of practice and >20 years of practice does not seem sufficient to this reviewer, since in <20 years of practice you can still have quite senior physicians. I would add an additional division: <10 years, 10-20 years, and >20 years of practice.

#### Minor Comments

4. How are the percentages calculated in Table 1? The percentages of every column should sum up to 100.

5. Could the authors comment on, if the physicians reported it in the questionnaire, which AI solutions they used in their daily life? Are they used in their personal life or in their work?

6. I assume there is an issue with the color legend for “Work facilitation” in Figure 2.

7. I would not only say that physicians think AI will not interfere with the number of appointments. A third of them thinks that AI solutions will increase the number of appointments.

8. I would include, if possible, a subanalysis of the responses per gender and discuss if there are any differences.

## Round 2 Review

### General Comments

This reviewer thanks the authors for the work done to improve the quality of the paper with this revision. However, I still have some comments.

### Specific Comments

#### Major Comments

1. In the previous review round, I asked about the AI solutions the health care workers used in their daily life. The authors replied by saying “the specific app (which uses AI algorithms) in their daily lives was not asked, but we believe it is the same as most of the people in Brazil: Instagram, WhatsApp, Waze, Google Apps, Alexa, Siri, Twitter and banks app.” This reviewer thinks this should be commented somewhere in the manuscript. From this questionnaire question, it seemed that workers have access to true AI solutions in their daily lives. However, these apps the authors mentioned as “AI solutions” use AI in their workflow but are not entirely based on AI and should not be considered “AI solutions.” Without commenting on this, the reader may think that the experience of this population in the use of AI is greater than it really is.

#### Minor Comments

2. I have not yet been able to access the supplementary material, and the color legend in Figure 2 is still not fixed.

3. In the text, it appears as *P*=.079, which is not significant. Please check.

4. The *P*=.0513 in Table 2 is not significant.

5. There should be a “Total” column in Table 1.

## Abbreviations

AI: artificial intelligence
